# Detection of *bla*_OXA-48_ and *mcr*-1 Genes in *Escherichia coli* Isolates from Pigeon (*Columba livia*) in Algeria

**DOI:** 10.3390/microorganisms10050975

**Published:** 2022-05-06

**Authors:** Lotfi Loucif, Widad Chelaghma, Esma Bendjama, Zineb Cherak, Meriem Khellaf, Asma Khemri, Jean-Marc Rolain

**Affiliations:** 1 Laboratoire de Biotechnologie des Molécules Bioactives et de la Physiopathologie Cellulaire (LBMBPC), Faculté des Sciences de la Nature et de la Vie, Université Batna 2, Batna 05000, Algeria; bendjamaesma@hotmail.fr (E.B.); cherak-zineb@hotmail.com (Z.C.); meriemkhellaf95@gmail.com (M.K.); asma.khemri95@outlook.fr (A.K.); 2 Département de Biologie, Université Abou Bekr Belkaid, Tlemcen 13000, Algeria; widadc2014@hotmail.com; 3 Département de Technologie Alimentaire, Institut des Sciences Vétérinaires et des Sciences Agronomiques, Université El Hadj Lakhder-Batna 1, Batna 05000, Algeria; 4 Faculté de Médecine et de Pharmacie, Aix Marseille Université, IRD, MEPHI, 13005 Marseille, France; jean-marc.rolain@univ-amu.fr; 5 IHU Méditerranée Infection, Marseille, Assistance Publique des Hôpitaux de Marseille, 13005 Marseille, France

**Keywords:** ESBL, OXA-48, *mcr-*1, *E. coli*, *Columba livia*, Algeria

## Abstract

The emergence and spread of β-lactams and colistin-resistant *Escherichia coli* in birds deserve a special concern worldwide. This study aimed to investigate the presence of β-lactams and colistin-resistant *Escherichia coli* strains isolated from the faeces of urban and rural pigeons in Batna, Algeria, and to characterise their molecular traits of resistance. Between March and April 2019, a total of 276 faecal droppings samples were collected in Batna, Algeria. Samples were subjected to selective isolation of β-lactams and colistin-resistant *Escherichia coli*. The representative colonies were then identified using Matrix-Assisted Laser Desorption-Ionization Time-of-Flight Mass Spectrometry. Antimicrobial susceptibility testing was performed using the disc diffusion method. β-lactamases, as well as *mcr* genes, were screened for by PCR and confirmed by sequencing. Genetic relatedness of the *mcr*-positive *E. coli* strains was determined using multi-locus sequence typing analysis. Transferability features of carbapenemase genes were assessed by conjugation experiments. Overall, thirty-five *E. coli* isolates were obtained only from urban pigeon samples. All carbapenem-resistant isolates harboured the *bla*_OXA-48_ gene as the only carbapenemase gene detected (n = 11), while *bla*_ESBL_ genes were detected in eighteen isolates. Out of the thirty-five isolates, four *E. coli* isolates were positive for the *mcr-*1 gene. The obtained *mcr-*1 positive *E. coli* isolates belonged to four STs, including ST1485, ST224, ST46, and a new ST. This study is the first to report the isolation of *E. coli* strains carrying the *mcr-*1 gene from pigeon faeces in Algeria and also the first to report the detection of *bla*_OXA-48_-positive *E. coli* in pigeons. Close surveillance is, therefore, urgently needed to monitor the dissemination of *bla*_OXA-48_ and *mcr-*1 producing *E. coli* strains in wildlife.

## 1. Introduction

Contaminated environments seem to be a leading factor in the dissemination of antibiotic resistance, as bacteria from various origins are able to mix and exchange antibiotic-resistance encoding genes [1,2]. Moreover, the role of the environment in promoting and spreading antibiotic-resistant bacteria and genes is understudied [3]. Birds are considered a good choice for monitoring urban ecosystems since they can be surveyed on a large scale, and they are easy to see and attractive to the population as well the fact that their occurrence and abundance are influenced by habitat characteristics. In addition, birds have also been postulated as potential reservoirs and vehicles of antibiotic resistance genes [4,5].

The *Columba livia* species of bird is common in cities in various countries and can transmit more than 30 diseases to humans via the air or their excreta [5]. Favourable environmental conditions, as well as the availability of food and the absence of predators, are the major factors implicated in the high increase in their populations in both urban and rural areas [6]. The presence of multi-drug resistant bacteria in pigeons is generally linked to faecal contamination of both human and animal origin [7]. Carriage of such bacteria in pigeon faeces has been reported in different countries, with much of the focus being on *Escherichia coli* species [8,9,10].

*Escherichia coli* is a Gram-negative bacterium which holds a special place in the microbiological world because some of them can cause severe infections in animals and humans, but they can also represent an important part of the autochthonous microbiota of many hosts. Of main concern is the possible transmission of resistant *E. coli* between animals and humans via various pathways, including direct contact, the food chain, or contact with animal excretion [11]. In the literature, numerous genes have been detected in *E. coli* species of human and animal origins that confer resistance to β-lactams (extended-spectrum β-lactamases and carbapenemase) and to colistin antibiotics. The increase in carbapenem and colistin-resistant bacteria is considered one of the most critical public health concerns since carbapenems and colistin are often used as a last-line treatment for multi-drug resistant Gram-negative bacterial infections [12,13]. The most emerged carbapenemase type worldwide is OXA-48-like enzyme variants, which are becoming the main carbapenemase type in *Enterobacteriaceae* worldwide, particularly in Mediterranean countries [14,15]. It was initially identified in a *K. pneumoniae* strain from a 54-year-old man with skin burns and a urinary tract infection from Istanbul, Turkey, in 2001 [16]. After the first identification, an outbreak of OXA-48-producing *K. pneumoniae* strains was described in Istanbul, Turkey, between May 2006 and January 2007, and since then, it has been rapidly diffused worldwide in different niches [14,17]. On the other hand, more recently, Liu et al. described the first plasmid-mediated colistin resistance mechanism, *mcr-*1, in human *K. pneumonia*e and *E. coli* recovered from provinces in China between April 2011 and November 2014 [18]. Currently, the *mcr-*1 gene has been found in different genera of the *Enterobacteriaceae* such as *Klebsiella*, *Escherichia,* and *Enterobacter* isolated from various sources, including animals and water samples, indicating that colistin resistance determinants have also disseminated into the environment notably both urban and rural areas [7,19]. To date, studies revealing the emergence of carbapenem and colistin-resistant bacteria isolated from pigeons are still limited.

Therefore, the aim of this study was to screen for the presence of ESBL, carbapenemase and *mcr*-producing *E. coli* isolates in faecal droppings samples from urban and rural pigeons in Batna, Algeria.

## 2. Materials and Methods

### 2.1. Sample Collection

Between March and April 2019, a total of 276 fresh faecal droppings samples from pigeons of the *Columba livia* species were collected at different urban area (the city of Batna) locations (n = 191), including a public park (n = 50), school (n = 50), university (n = 15), mosque (n = 2), different household residences: 800 household residences (n = 6), 1020 household residences (n = 24), 126 household residences (n = 23), 74 household residences (n = 1), nearest to a sewage treatment plant (n = 20), and as well as rural area (from animal farms (n = 85) in EL Madher locality) in Batna, eastern Algeria. Fresh faecal droppings samples were collected aseptically in sterile containers and were immediately transferred at 4 °C to the laboratory for analysis. The samples were first pooled before the isolation procedure, where each pooled sample contained two, three or five samples of the same location.

### 2.2. β-Lactams and Colistin-Resistant-E. coli Isolation and Bacterial Identification

The isolation of extended-spectrum-cephalosporins, carbapenems and colistin-resistant-*E. coli* started with a selective enrichment step in brain-heart infusion (BHI) broth with 64 μg/mL vancomycin and supplemented with one of the four different selective antibiotics as follows: (1): 2 μg/mL cefotaxime, (2): 2 μg/mL ertapenem, (3): 9 μg/mL imipenem or (4): 3 μg/mL colistin, respectively. After overnight incubation at 37 °C, ten microliters were taken from the enrichment tubes and were inoculated into selective MacConkey agar plates with the same selective antibiotic combinations [20,21]. The bacterial identification of the obtained isolates was performed by Matrix-Assisted Laser Desorption Ionization-Time of Flight mass spectrometry (MALDI-TOF MS), as previously described [22].

### 2.3. Antimicrobial Susceptibility Testing

Antimicrobial drug susceptibility of the obtained isolates was determined on Mueller–Hinton agar using the standard disc diffusion method, as recommended by the Antibiogram Committee of the French Society for Microbiology (CA-SFM, 2019) (https://www.sfm-microbiologie.org/wp-content/uploads/2019/02/CASFM2019_V1.0.pdf; accessed on 1 March 2019). The obtained isolates were tested for antibiotic resistance using a panel of thirteen antibiotics, including amoxicillin (20 μg), cefoxitin (30 μg), ceftazidime (30 μg), cefotaxime (30 μg), cefepime (30 μg), aztreonam (30 μg), amoxicillin-clavulanic acid (20–10 μg), ertapenem (10 μg), imipenem (10 μg), tobramycin (10 μg), gentamicin (10 μg), amikacin (30 μg), and ciprofloxacin (5 μg). The *E. coli* ATCC 25922 strain was used for quality control assays. The results were interpreted according to the CA-SFM, 2019, as well as the Clinical and Laboratory Standards Institute (CLSI, 2017) breakpoints.

The minimal inhibitory concentration (MIC) of colistin was performed by broth microdilution applying the criteria of the European Committee on Antimicrobial Susceptibility Testing Guidelines, 2017 (https://www.eucast.org/; accessed on 15 May 2019).

### 2.4. Phenotypic Detection of Extended Spectrum β-Lactamase and Carbapenemase Production

The detection of ESBL was further performed phenotypically using the double-disk diffusion method (DDST), while the phenotypic investigation of carbapenemase production was performed using the modified carba NP (MCNP) test as previously described [23].

### 2.5. Molecular Detection of β-Lactamases and mcr Genes

The obtained strains were tested for the presence of extended-spectrum β-lactamases (*bla*_SHV_, *bla*_TEM_, *bla*_CTX-M_) and for the most common carbapenemase genes (*bla*_KPC_, *bla*_NDM_, *bla*_VIM_, and *bla*_OXA-48_-like) using real-time PCR (qPCR) with specific primers (Table 1). The colistin resistance gene (*mcr-*1, *mcr-*2, *mcr-*3, *mcr-*4, *mcr-*5, and *mcr-*8) was also searched for by qPCR [24,25,26]. Standard PCR and sequencing of the positive real-time PCR strains harbouring the carbapenemase or *mcr* genes were also performed.

### 2.6. Conjugation Experiment

The transferability of carbapenemase genes was determined through a conjugation experiment (broth mating method) using an azide-resistant *E. coli* J53 recipient strain and two donor strains. The transconjugants were selected on nutrient agar containing ertapenem (2 µg/mL) and sodium azide (200 µg/mL) [20]. The obtained transconjugants were verified by antimicrobial drug susceptibility testing and the modified carba NP test and were confirmed to have the *bla*_OXA−48_ gene by PCR.

### 2.7. Multilocus Sequence Typing

To determine the epidemiological relationships, MLST analysis was carried out on the *mcr-*1-positive *E. coli* isolates. Multilocus sequence typing (MLST) was performed by targeting seven housekeeping genes (*adk*, *fumC*, *gyrB*, *icd*, *mdh*, *purA*, and *recA*) [31]. The obtained sequences were analysed through an *E. coli* MLST database website (http://mlst.warwick.ac.uk/mlst/dbs/Ecoli; accessed on 15 October 2020).

### 2.8. Statistical Analysis

The isolation rate of the targeted drug resistant-*E. coli* (ESBL, carbapenemase and *mcr-*1-positive isolates) related to the sampling sites was analysed by performing the Pearson chi-square test using SPSS (version 26.0; SPSS, Inc., Chicago, IL, USA). The level of significance was set at a *p*-value < 0.05.

## 3. Results

### 3.1. Bacterial Identification and Antimicrobial Susceptibility Testing

Thirty-five *E. coli* isolates were identified from pigeon faeces recovered from the different urban areas, including the university (5.71%), 1020 household residences (5.71%), 800 household residences (8.57%), a public park (17.15%), and thosenearest to a sewage treatment plant (62.86%). However, no *E. coli* isolates were obtained from rural samples as well as other urban areas (school, mosque, 126 household residences, 74 household residences). In this context, thePearson chi-square test revealed no significant effect of the sampling site on the rate of positive isolated strains (positivity) (χ^2^ = 30; *p* = 0.314).

**Figure 1:**
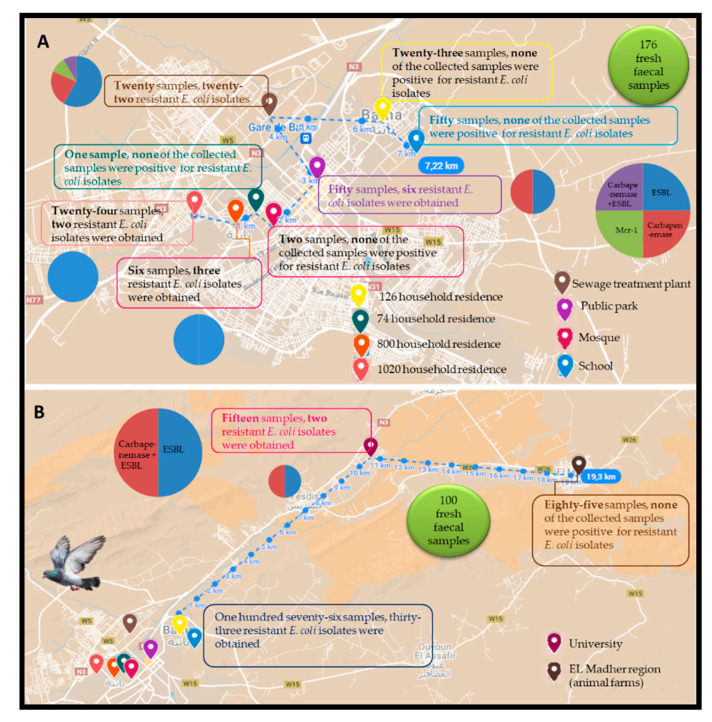
Location of sampling points with the detection rate of AMR *E. coli* in the different sites targeted in this study. (**A**) 176 fresh faecal samples; (**B**) 100 fresh faecal samples.

All the obtained isolates were resistant to amoxicillin (n = 35), however overall, twenty-four were resistant to amoxicillin-clavulanic acid (n = 24), followed by cefotaxime (n = 23), ceftazidime (n = 18), cefepime (n = 15), ertapenem (n = 14), aztreonam (n = 10), and cefoxitin (n = 2). Resistance to ciprofloxacin, tobramycin, and gentamicin was observed in twenty-two, ten, and seven isolates, respectively. Imipenem and amikacin showed excellent antibacterial activity against the obtained isolates with a susceptibility of 100%. In addition, four *E. coli* isolates were resistant to colistin with minimum inhibitory concentration measured at 4 µg/mL. The location of sampling points with the detection rate of AMR *E. coli* in various categories of sites targeted in this study was generated using Google Maps with open data (https://www.google.dz/maps; accessed on 15 December 2021) and presented in Figure 1.

### 3.2. Molecular Detection of ESBL, Carbapenemase and mcr Genes

The genotyping results of ESBL, carbapenemase and *mcr* genes among the obtained isolates are shown in Table 2. Of the 35 isolates, eighteen were ESBL producers, seven were carbapenemase producers, and four isolates were positive for carbapenemase and other β-lactamase types. Among the eighteen ESBL producing isolates, ten were found to be positive for the combination *bla*_CTX-M−A_ and *bla*_TEM_ gene, while eight of the obtained isolates were positive for the *bla*_CTX-M−A_ gene. The eleven carbapenemase-producing isolates were *bla*_OXA-48_ positive. Among the *bla*_OXA-48_ positive *E. coli*, two isolates harboured the *bla*_OXA-48_, *bla*_CTX-M−A_, and *bla*_TEM_ genes, one isolate of each was positive for the combination *bla*_OXA-48_ and *bla*_TEM_ or *bla*_OXA-48_, *bla*_CTX-M−A_ genes, respectively. The remaining seven isolates harboured only the *bla*_OXA-48_ gene. Out of 35 isolates, four *E. coli* isolates were positive for the *mcr-*1 gene.

### 3.3. Conjugation Experiment

Our study showed that the two tested *E. coli* isolates that carried the *bla*_OXA-48_ gene were successfully transferred to *E. coli* J53. The antimicrobial susceptibility of the obtained transconjugants (TCP21 and TCP30) showed that they were resistant to amoxicillin-clavulanic acid and ertapenem and were positive for the MCNP test. PCR results confirmed the presence of the *bla*_OXA-48_ gene in the two obtained transconjugants.

### 3.4. Multilocus Sequence Typing

MLST results showed that the four *mcr-*1 positive*-E. coli* isolates belonged to four different sequence types, including ST1485, ST224, ST46, and new ST.

## 4. Discussion

Around the world, there are significant numbers of pigeons living in close contact with humans and other animals in rural and urban areas [32]. Various studies on the contamination levels of pigeon faeces in public areas revealed that pigeon faeces represent a source of various zoonotic agents for humans and animals and have been identified as a potential source and vector that can spread antibiotic-resistant bacteria and genes [33,34,35,36,37]. In this regard, the emergence of extended-spectrum cephalosporins, carbapenem, and colistin resistance in pigeon faeces is a serious challenge worldwide, in both urban and rural areas. In our study, we report the detection of *bla*_ESBL_, *bla*_OXA-48_, and *mcr-*1 genes from urban pigeon faeces in Algeria. These results can be explained by different contributing factors, including diverse feeding habits of urban pigeons such as sewage treatment plants and municipal solid waste dumping grounds [38]. These feeding habits of urban pigeons could lead to them being contaminated with medically important bacteria or residual antimicrobials and chemicals since they may rely on waste or nearby refuse containers as food sources [5,39]. In addition, various authors have suggested that pigeons can interact with other birds, which would facilitate the acquisition and dissemination of this resistance to other species [5].

The carriage of ESBL producers in pigeons in our study was comparable to previous studies around the world, which have reported the detection of *E. coli* isolates harbouring *bla*_CTX-M_ genes from pigeons, including Bangladesh, France, Germany, Nicaragua, China, and Brazil [9,10,34,35,40,41]. In this study, we also detected the *bla*_OXA-48_ gene in *E. coli* isolates from different urban places around the city, including a sewage treatment plant, a university, and a public park. To the best of our knowledge, the two last urban areas are among the most dynamic areas in the city. The public park is the most urbanised, popular, and economically active region in the city of Batna, and it is located in an area marked by urban sprawl and overcrowding. *Columba livia* might favour the inter-genus or inter-species horizontal propagation of antibiotic-resistance genes because their faeces can harbour different resistant bacteria representative of the various environments that the birds recently visited [39]. Domestic pigeons do not travel long distances (maximum 5.29 km), and they have to meet their needs with what they find within the signalled distance. In this study, the sampled areas were located in environments with a high human population, close to hospitals and wastewater, known reservoirs of antibiotic-resistance genes, where the birds can access water and food that is contaminated with pharmaceutical products such as antibiotics. In this context and in the same city where our study was conducted, the first detection of the p*bla*_OXA-48_ gene was described in 2014 at Batna university hospital, then from migratory birds, community-acquired infection, currency, and more recently from hospital wastewater [20,21,42,43,44], suggesting that the detected genes in pigeon are related to their feeding mode in different locations including hospitals and wastewater or by contact with other birds such as migratory birds. From pigeons, only two studies have reported the detection of carbapenemase-producing Gram-negative bacteria worldwide. The first such study was conducted on pigeon faeces collected in Algeria and France, where the authors identified the presence of carbapenemases-encoding genes in 16 out of the 73 studied samples (13 were positive for *bla*_OXA-58_, 12 *bla*_OXA-51-like_, and eight carried the *bla*_OXA-23_ genes) [33]. The second report detected *bla*_MUS-2_, a novel variant of the chromosome-encoded *bla*_MUS-1_ associated with carbapenem resistance in *Myroides odoratimimus* isolates in Lebanon [45]. Importantly, in this study, we report the first detection of the *mcr-*1 gene in pigeons in Algeria, where colistin is considered a drug of last resort for human medicine for the treatment of infections caused by Gram-negative bacteria. However, there have only been a few reports of the *mcr* gene in pigeons. In agreement with our findings, a study in Qatar reported that only one *E. coli* isolate from pigeon faecal samples harboured the *mcr-*1 gene [8]. *mcr-*1 and *mcr-*3 genes have also been detected in China, with a prevalence of 13.1% and 5.1%, respectively [46]. Another study conducted in China signalled the detection of *mcr-*4 and *mcr-*5 genes with a prevalence of 17.2% and 3%, respectively [47]. In our study, the detection of the *mcr-*1 gene has been reported only in *E. coli* isolates obtained from faecal droppings samples collected near sewage treatment plants. This result can be explained by the feeding habits of urban pigeons, where a recent study has reported the detection of the *mcr-*1 gene with the same STs from the sewage treatment plants where our analysed pigeons fed (unpublished data), indicating that the surrounding environment could be the origin of the detected resistance genes. To the best of our knowledge, wastewater from different hospitals could be discharged into the sewage treatment plants inviting the possibility that the reported genes could be related to the hospital settings. No *mcr-*1 genes were found in the sampled rural area (animal farms), which may be explained by the limited or prudent use of antibiotics, particularly colistin.

The MLST results showed that the four *mcr-*1 positive *E. coli* isolates belonged to four different sequence types, including ST1485, ST224, ST46, and a new ST. In fact, these STs appear to be well adapted to animals living in rural and urban areas and have been reported worldwide, mostly in association with plasmid-mediated *bla*_CTX-M_-type genes or, similar to our study, with the *mcr-*1 gene. The ST224 has already been reported in isolates with the *bla*_CTX-M_ gene from cats in France and Brazil [48,49], from food-producing animals (buffalo calves) in Brazil [50], and from a deer in Spain [51]. In addition, the ST224 has been reported in strains with colistin resistance from chicken meat in Algeria [52]. ST1485 *E. coli* isolate has already been isolated from rural dogs in Spain [53] and from birds in Chile (Andean condors) [54]. Similarly, ST46 has been previously reported in an *E. coli* strain with the *mcr-*1 gene from chicken faeces and in pets in China [55,56] and with CTX-M type ESBL from pig samples from Nigeria [57]. This suggests that pigeons could facilitate the crossover of antimicrobial resistance with other animals in the local region and contribute to the further spread of these resistance genes.

## 5. Conclusions

To conclude, we report here for the first time the presence of the *mcr-*1 gene in pigeon droppings in Algeria and also report the first detection of OXA-48-producing *E. coli* in pigeon droppings. This study clearly illustrates that pigeons, which live in close proximity to humans, could play a role as potential reservoirs of multi-drug-resistant bacteria, including carbapenemase and *mcr* producers in urban areas. Hence, risk management measures should be undertaken to limit the emergence and spreading of AMR in Algeria.

In light of these data, future studies should be conducted to identify multi-drug-resistant bacteria transmission pathways in order to understand the potential role of such birds in the spread of carbapenemase and *mcr-*1 genes.

## Figures and Tables

**Table 1:** Oligonucleotide primers and probes used for polymerase chain reaction.

Type of PCR	Primers	Primer Sequence (5′->3′)	References
Real-time PCR	TEM-F.	GCATCTTACGGATGGCATGA	[27]
	TEM-R	GTCCTCCGATCGTTGTCAGAA	
	TEM-probe	6-Fam CAGTG CTGCCATAACCA TGAGTGA-BHQ-1	
Real-time PCR	SHV-F	TCCCATGATGAGCACCTTTAAA	
	SHV-R	TCCTGCTGGCGATAGTGGAT	
	SHV-probe	Cy5-TGCCGGTGACGAACAGCTGGAG-BBQ-650	
Real-time PCR group A	CTX-A-F	CGGGCRATGGCGCARAC	
	CTX-A-R	TGCRCCGGTSGTATTGCC	
	CTX-A-probe	Yakima Yellow-CCARCGGGCGCAGYTGGTGAC-BHQ1	
Real-time PCR group B	CTX-B-F	ACCGAGCCSACGCTCAA	
	CTX-B-R	CCGCTGCCGGTTTTATC	
	CTX-B-probe	Yakima Yellow- CCCGCGYGATACCACCACGC-BHQ1	
Real-time PCR	KPC-F	GATACCACGTTCCGTCTGGA	[28]
	KPC-R	GGTCGTGTTTCCCTTTAGCC	
	KPC-Probe	6-FAM-CGCGCGCCGTGACGGA AAGC-TAMRA	
Real-time PCR	VIM-F	CACAGYGGCMCTTCTCGCGGAGA	
	VIM-R	GCGTACGTYGCCACYCCAGCC	
	VIM-Probe	6-FAM-AGTCTCCACGCACTTTCATGA CGACCGCGTCGGCG-TAMR	
Real-time PCR	NDM-F	GCGCAACACAGCCTGACTTT	[29]
	NDM-R	CAGCCACCAAAAGCGATGTC	
	NDM-Probe	6-FAM-CAACCGCGCCCAACTTTGGC-TAMRA	
Real-time PCR	OXA48-RT-F	TCTTAAACGGGCGAACCAAG	[28]
	OXA48-RT-R	GCGTCTGTCCATCCCACTTA	
	OXA48-RT-Probe	6-FAM-AGCTTGATCGCCCTCG ATTTGG-TAMRA	
Standard PCR	OXA-48-F	TTGGTGGCATCGATTATCGG	[30]
	OXA-48-R	GAGCACTTCTTTTGTGATGGC	
Real-time PCR	*mcr-*1–2-F	CTGTGCCGTGTATGTTCAGC	[25]
	*mcr-*1–2-R	TTATCCATCACGCCTTTTGAG	
	Probe (*mcr-*1–2)	FAM-TATGATGTCGATACCGCCAAATACC-TAMRA	
	Probe (*mcr-*2)	VIC-TGACCGCTTGGGTGTGGGTA-TAMRA	
Standard PCR	*mcr-*1-F	GCAGCATACTTCTGTGTGGTAC	[24]
	*mcr-*1-R	TATGCACGCGAAAGAAACTGGC	
Real-time PCR	*mcr-*3-F	TGAATCACTGGGAGCATTAGGGC	[25]
	*mcr-*3-R	TGCTGCAAACACGCCATATCAAC	
	*mcr-*3-probe	FAM-TGCACCGGATGATCAGACCCGT-TAMRA	
Real-time PCR	*mcr-*4-F	GCCAACCAATGCTCATACCCAAAA	
	*mcr-*4-R	CCGCCCCATTCGTGAAAACATAC	
	*mcr-*4-probe	FAM-GCCACGGCGGTGTCTCTACCC-TAMRA	
Real-time PCR	*mcr-*5-F	TATCCCGCAAGCTACCGACGC	
	*mcr-*5-R	ACGGGCAAGCACATGATCGGT	
	*mcr-*5-probe	FAM-TGCGACACCACCGATCTGGCCA-TAMRA	
Real-time PCR	*mcr-*8-F	TCCGGGATGCGTGACGTTGC	[26]
	*mcr-*8-R	TGCTGCGCGAATGAAGACGA	
	*mcr-*8-probe	FAMTCATGGAGAATCGCTGGGGGAAAGC-TAMRA	

**Table 2:** Antibiotic susceptibility testing, resistance genes, and sequence types of the *E. coli* isolates obtained in this study.

Strains	Medium	Site	Antibiotic Resistance Genes												Phenotypic Detection of β-Lactamases		Antibiotic Resistance Genes	ST
			FOX	CTX	CAZ	FEP	ATM	AMC	ETP	IMP	TOB	GN	AK	CIP	DDST MCNP Test			
**P1**	CTX	PP	S	R	R	R	R	S	S	S	S	S	S	R	P	N	*bla*_CTX-M-A_, *bla*_TEM_	ND
**P2**	ETP	PP	I	S	S	S	S	R	R	I	S	S	S	R	N	P	*bla*_OXA-48_	ND
**P3**	ETP	PP	S	S	S	S	S	R	R	I	S	S	S	R	N	P	*bla*_OXA-48_	ND
**P4**	ETP	PP	I	S	S	S	S	R	R	I	S	S	S	R	N	P	*bla*_OXA-48_	ND
**P5**	CTX	PP	S	R	R	R	R	R	R	S	S	S	S	S	P	N	*bla*_CTX-M-A_, *bla*_TEM_	ND
**P6**	CTX	PP	S	R	S	I	I	R	S	S	R	R	S	R	P	N	*bla*_CTX-M-A_	ND
**P7**	CTX	800D	S	R	S	R	R	S	S	S	S	S	S	R	P	N	*bla*_CTX-M-A_	ND
**P8**	CTX	800D	S	R	R	R	R	R	S	S	R	S	S	R	P	N	*bla*_CTX-M-A_, *bla*_TEM_	ND
**P9**	CTX	800D	S	R	R	R	R	S	S	S	S	S	S	R	P	N	*bla*_CTX-M-A_	ND
**P10**	CTX	UNIV	R	R	R	S	I	R	R	S	I	R	S	S	P	P	*bla*_OXA-48_, *bla*_CTX-M-A_, *bla*_TEM_	ND
**P11**	CTX	UNIV	S	R	R	R	R	S	S	S	S	S	S	S	P	N	*bla*_CTX-M-A_, *bla*_TEM_	ND
**P12**	IMP	1020D	S	R	R	R	R	S	S	S	S	S	S	S	P	N	*bla*_CTX-M-A_, *bla*_TEM_	ND
**P13**	IMP	1020D	S	R	S	R	S	S	S	I	S	S	S	R	P	N	*bla*_CTX-M-A_	ND
**P14**	CTX	NSTP	S	R	R	R	R	R	S	S	R	S	S	R	P	N	*bla*_CTX-M-A_, *bla*_TEM_	ND
**P15**	CTX	NSTP	S	R	R	R	R	S	S	S	I	S	S	S	P	N	*bla*_CTX-M-A_, *bla*_TEM_	ND
**P16**	ETP	NSTP	S	S	I	S	S	R	R	I	S	S	S	S	N	P	*bla*_OXA-48_	ND
**P17**	ETP	NSTP	S	R	R	S	S	R	R	I	S	S	S	S	N	P	*bla*_OXA-48_	ND
**P18**	ETP	NSTP	S	R	R	S	S	R	R	I	R	R	S	S	N	P	*bla*_OXA-48_	ND
**P19**	ETP	NSTP	S	S	S	S	S	R	R	S	R	R	S	I	N	P	N	ND
**P20**	CTX	NSTP	S	R	S	S	S	R	S	S	R	S	S	S	P	N	*bla*_CTX-M-A_	ND
**P21**	ETP	NSTP	S	R	R	S	I	R	R	I	R	R	S	S	P	P	*bla*_OXA-48_, *bla*_CTX-M-A_, *bla*_TEM_	ND
**P22**	CTX	NSTP	R	R	R	S	S	R	S	S	S	S	S	S	P	N	*bla*_CTX-M-A_, *bla*_TEM_	ND
**P23**	CTX	NSTP	S	R	I	I	R	R	S	S	R	S	S	R	P	N	*bla*_CTX-M-A_	ND
**P24**	CTX	NSTP	S	R	R	R	I	R	S	S	S	S	S	R	P	N	*bla*_CTX-M-A_, *bla*_TEM_	ND
**P25**	ETP	NSTP	S	I	R	I	S	R	R	I	I	R	S	R	N	P	*bla*_OXA-48_, *bla*_TEM_	ND
**P26**	ETP	NSTP	S	S	S	S	S	R	R	I	S	S	S	R	N	P	N	ND
**P27**	CTX	NSTP	S	R	R	R	S	R	S	S	R	S	S	R	P	N	*bla*_CTX-M-A_	ND
**P28**	CTX	NSTP	S	R	R	R	I	S	S	S	S	S	S	R	P	N	*bla*_CTX-M-A_	ND
**P29**	CTX	NSTP	S	R	I	R	S	R	S	S	S	S	S	S	P	N	*bla*_CTX-M-A_, *bla*_TEM_	ND
**P30**	CTX	NSTP	S	R	R	R	I	R	R	S	S	S	S	R	P	P	*bla*_OXA-48_, *bla*_CTX-M-A_	ND
**P31**	ETP	NSTP	S	I	I	S	S	R	R	I	S	S	S	R	N	P	*bla*_OXA-48_	ND
**P32**	COL	NSTP	S	S	S	S	S	S	S	S	S	S	S	R	N	N	*mcr-*1	1485
**P33**	COL	NSTP	S	S	S	S	S	R	S	S	R	R	S	R	N	N	*mcr-*1	224
**P34**	COL	NSTP	S	S	S	S	S	R	S	S	S	S	S	R	N	N	*mcr-*1	46
**P35**	COL	NSTP	S	S	S	S	S	S	S	S	S	S	S	R	N	N	*mcr-*1	New ST

PP: public park, univ: university, 800D: 800 household residence, 1020: 1020 household residence, NDTP: nearest to sewage treatment plant, AX: amoxicillin, FOX: cefoxitin, CTX: cefotaxime, CAZ: ceftazidime, FEP: cefepime, ATM: aztreonam, AMC: amoxicillin/clavulanate, ETP: ertapenem, IMP: imipenem, TOB: tobramycin, CN: gentamicin, CIP: ciprofloxacin, COL: colistin, R: resistant, I: intermediate, S; sensible, ST: sequence type, N: negative, P: positive, DDST: double-disk diffusion method, MCNP test: modified carba NP test.

## Data Availability

The data presented in this study are available on request from the corresponding author.
